# A novel monoclonal antibody targeting coxsackie virus and adenovirus receptor inhibits tumor growth *in vivo*

**DOI:** 10.1038/srep40400

**Published:** 2017-01-11

**Authors:** Manabu Kawada, Hiroyuki Inoue, Masunori Kajikawa, Masahito Sugiura, Shuichi Sakamoto, Sakiko Urano, Chigusa Karasawa, Ihomi Usami, Mitsuru Futakuchi, Tohru Masuda

**Affiliations:** 1 Institute of Microbial Chemistry (BIKAKEN), Numazu, Microbial Chemistry Research Foundation, 18-24 Miyamoto, Numazu-shi, Shizuoka 410-0301, Japan; 2 Institute of Microbial Chemistry (BIKAKEN), Laboratory of Oncology, Microbial Chemistry Research Foundation, 3-14-23 Kamiosaki, Shinagawa-ku, Tokyo 141-0021, Japan; 3 Medical & Biological Laboratories Co., Ltd., 4-5-3 Sakae, Naka-ku, Nagoya, Aichi 460-0008, Japan; 4 Department of Molecular Toxicology, Nagoya City University Graduate School of Medical Sciences, 1 Kawasumi, Mizuho-cho, Mizuho-ku, Nagoya 467-8601, Japan

## Abstract

To create a new anti-tumor antibody, we conducted signal sequence trap by retrovirus-meditated expression method and identified coxsackie virus and adenovirus receptor (CXADR) as an appropriate target. We developed monoclonal antibodies against human CXADR and found that one antibody (6G10A) significantly inhibited the growth of subcutaneous as well as orthotopic xenografts of human prostate cancer cells *in vivo*. Furthermore, 6G10A also inhibited other cancer xenografts expressing CXADR, such as pancreatic and colorectal cancer cells. Knockdown and overexpression of CXADR confirmed the dependence of its anti-tumor activity on CXADR expression. Our studies of its action demonstrated that 6G10A exerted its anti-tumor activity primarily through both antibody-dependent cellular cytotoxicity and complement-dependent cytotoxicity. Moreover, 6G10A reacted with human tumor tissues, such as prostate, lung, and brain, each of which express CXADR. Although we need further evaluation of its reactivity and safety in human tissues, our results show that a novel anti-CXADR antibody may be a feasible candidate for cancer immunotherapy.

Antibodies are a promising strategy for anti-tumor treatment because their effect is selective and potent against cancer cells. Recently, blockade of immune checkpoints using anti-PD-1 antibody was shown to be a promising approach in some cancer patients^1^. Many antibodies have been developed against molecular targets and used in clinical settings. However, molecular targets for antibody development are getting exhausted. In order to create new antibodies against a new molecular target, we used a method termed SST-REX (signal sequence trap by retrovirus-mediated expression screening)^2^. SST-REX fuses cDNAs of proteins that contain a signal sequence, similar to what is found in membrane-bound or secreted proteins, with the thrombopoietin receptor (MPL) gene. The fusion genes are then transfected into inteleukin-3 (IL-3)-dependent Ba/F3 cells and then clones that can survive without IL-3 are selected^3^,^4^. Furthermore, the Ba/F3 cells can be used to immunize mice to create antibodies. Thus, the SST-REX method is an approach that can be used to create antibodies against membrane-bound or secreted proteins.

Human androgen-dependent prostate cancer LNCaP cells make palpable tumors only when injected into nude mice^5^,^6^. We previously established a highly tumorigenic subline, LNCaP-CR cells, from parental LNCaP cells by the selection in cytokine-induced apoptosis resistance^5^,^6^. LNCaP cells were killed by the treatment with IL-1β and IL-6, but LNCaP-CR cells were not^5^. We then used these cell lines and SST-REX to identify a molecular target appropriate for cancer chemotherapy. As a result, we found that LNCaP-CR cells express higher levels of CXADR than LNCaP cells.

There are few studies about the role of CXADR in cancer biology. Saito *et al*. reported that CXADR is a critical regulator of the survival and growth of oral squamous cancer cells^7^. On the other hand, some papers have shown that CXADR suppresses the growth of colorectal and gastric cancer cell lines^8^,^9^. Thus, the function of CXADR in cancer development is controversial. We previously reported the strategy for the construction of antibodies against CXADR and the preliminary results of their anti-tumor effects in EORTC-NCI-AACR Symposium on Molecular Targets and Cancer Therapeutics^10^. In this paper, we fully describe the development of a novel CXADR antibody and its anti-tumor activity *in vitro* and *in vivo* in detail.

## Results

### Development of mouse monoclonal antibodies against CXADR

**Figure 1:**
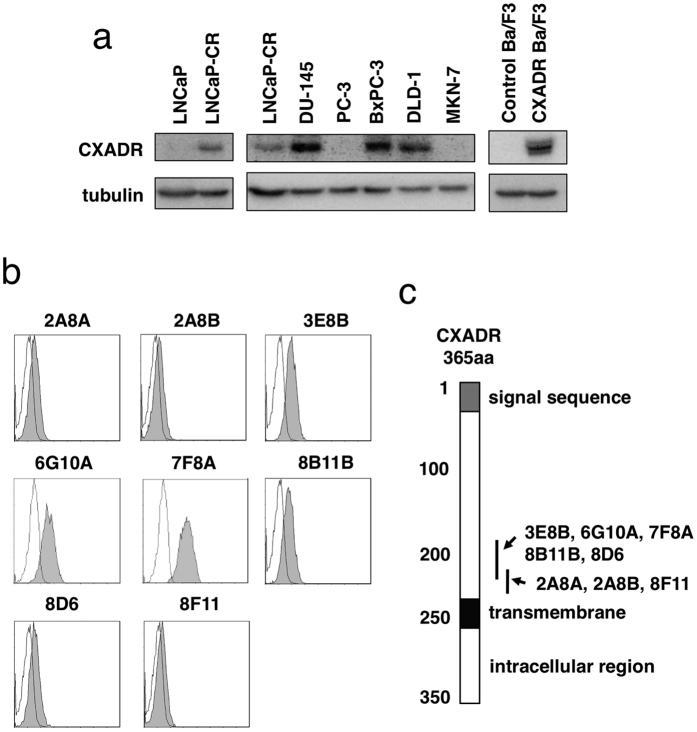
CXADR expression in various cell lines and development of anti-CXADR antibodies. (**a**) Expression of CXADR and tubulin in the indicated cell lines was detected by Western blotting. The gels have been run under the same experimental conditions and cropped to show protein bands corresponding to CXADR or tubulin as indicated. (**b**) Ba/F3 cells expressing human CXADR were incubated with the indicated antibody clones (gray) or isotype control antibodies (white), followed by by flow cytometry. (**c**) The positions in CXADR that are bound by the indicated antibody clones are shown.

Using SST-REX method we isolated 207 and 150 clones of Ba/F3 cells survived without IL-3 from LNCaP-CR and LNCaP cells, respectively. After sequencing the cDNA fused with the MPL gene in the isolated Ba/F3 clones, we obtained 67 and 50 candidate genes from LNCaP-CR and LNCaP cells, respectively. Among them, we evaluated 10 molecules that were expressed preferentially in LNCaP-CR cells compared to LNCaP cells. We chose CXADR as a target for further studies because there were few reports about the role of CXADR on cancer development^7^. As shown in Fig. 1a, LNCaP-CR cells expressed higher levels of CXADR than LNCaP cells. In respect to other prostate cancer cell lines, DU-145 human androgen-independent prostate cancer cells also expressed CXADR, but PC-3, another androgen-independent prostate cancer cell line, did not (Fig. 1a).

We immunized normal BALB/c mice having normal immune responses with Ba/F3 cells that expressed CXADR (Fig. 1a) in order to create mouse monoclonal antibodies against CXADR (Fig. 1b,c). We identified eight antibodies that bound CXADR proteins on the surface of Ba/F3 cells (Fig. 1b), but they recognized different regions of CXADR (Fig. 1c). All antibodies we isolated failed to detect mouse CXADR (data not shown). Determination of the specific CXADR epitopes the antibodies bound to is described in the Materials and Methods.

### Anti-CXADR antibodies display anti-tumor activity against LNCaP-CR cells *in vivo*

**Figure 2:**
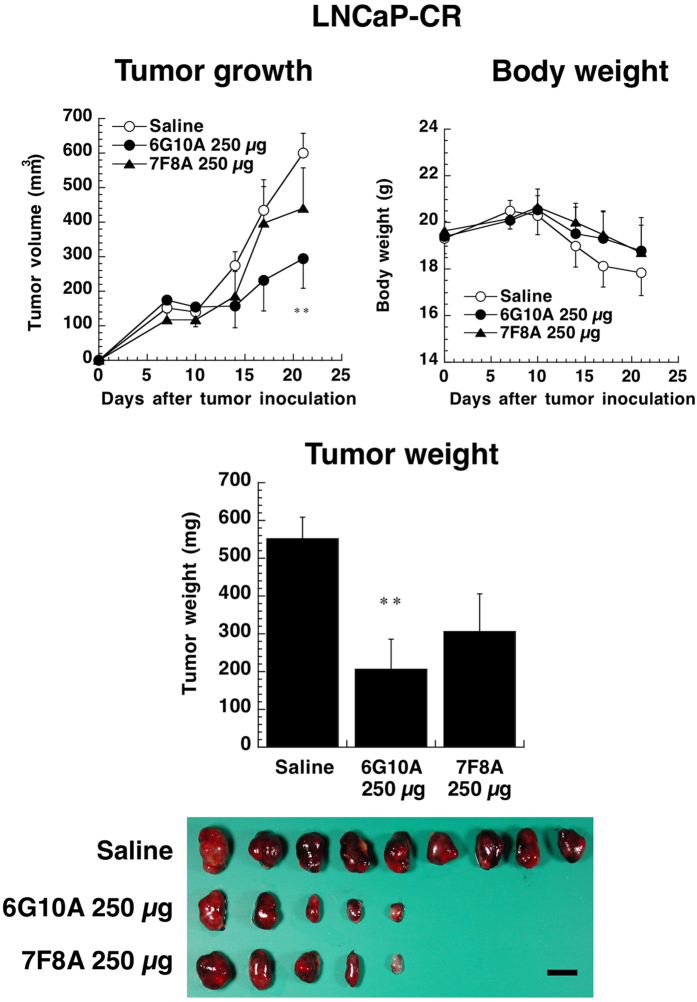
Effect of anti-CXADR antibodies on the growth of LNCaP-CR subcutaneous tumors *in vivo*. LNCaP-CR cells were injected subcutaneously into male nude mice. The indicated antibodies (250 μg/day) were administered intravenously 1, 7, and 14 days after cancer cell injection. Mice were sacrificed 21 days after the cancer cell injection, and the LNCaP-CR tumors were excised and weighed. The values are means ± SEM (n = 5). **P < 0.01 versus the control values. Scale bar is 1 cm.

Although anti-CXADR antibodies were added to the culture of LNCaP-CR cells, all antibodies did not affect the growth of LNCaP-CR cells directly *in vitro* (Supplementary Fig. 1). We next examined the anti-tumor activity of anti-CXADR antibodies *in vivo* using xenograft models. LNCaP-CR cells were inoculated subcutaneously into nude mice, and anti-CXADR antibodies were injected intravenously every day for 11 days. Clones 7F8A and 6G10A inhibited the growth of xenograft LNCaP-CR tumor cells (Supplementary Fig. 2). We next evaluated the anti-tumor activities of clones 6G10A and 7F8A in greater detail using highly purified antibodies. As shown in Fig. 2, clone 6G10A significantly inhibited the growth of LNCaP-CR tumors *in vivo,* even when the antibody injections were decreased to single weekly administrations without any adverse effects on the host mice. Clone 7F8A did not significantly inhibit tumor growth *in vivo* (Fig. 2). Although clone 6G10A inhibited LNCaP-CR tumors in a dose-dependent manner (Supplementary Fig. 3), 250 μg/mouse had the greatest *in vivo* anti-tumor effect, and this was the dose that we used for our subsequent studies. Clone 6G10A was able to inhibit the growth of LNCaP-CR tumors even when administration was initiated as late as 14 days after cancer cell inoculation (Supplementary Fig. 4).

### Anti-CXADR antibody clone 6G10A exerts anti-tumor activity on orthotopic LNCaP-CR xenografts

**Figure 3:**
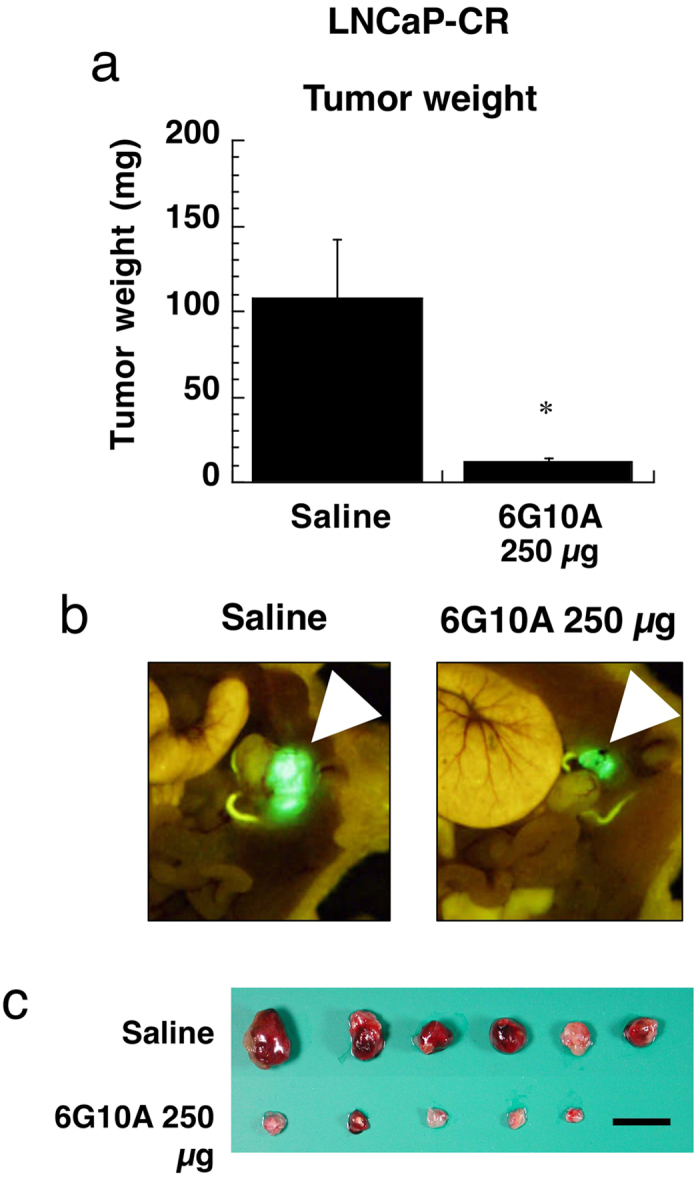
Effect of anti-CXADR antibody 6G10A on the growth of orthotopic LNCaP-CR tumors *in vivo*. LNCaP-CR cells were injected orthotopically in the prostate of male nude mice. Antibodies (250 μg/day) were administered intravenously 1, 7, and 14 days after the cancer cell injection. Mice were sacrificed 21 days after the cancer cell injection, and the LNCaP-CR tumors were excised and weighed. The values are means ± SEM (n = 5). *P < 0.05 versus the control values. (**a**) Tumor weight. (**b**) Representative photos of the murine prostate. Arrowheads indicate GFP-transfected LNCaP-CR tumors. (**c**) Excised tumors. Scale bar is 1 cm.

We next examined the anti-tumor effect of 6G10A on the growth of LNCaP-CR cells implanted orthotopically into the murine prostate *in vivo*. We found that 6G10A antibodies almost completely inhibited the growth of orthotopic xenograft LNCaP-CR tumors even with once-a-week administrations (Fig. 3). These results clearly demonstrate the potency and good distribution of 6G10A antibody into the target organs.

### Anti-CXADR antibody clone 6G10A exerts anti-tumor effects against CXADR-expressing cancer cells *in vivo*

**Figure 4:**
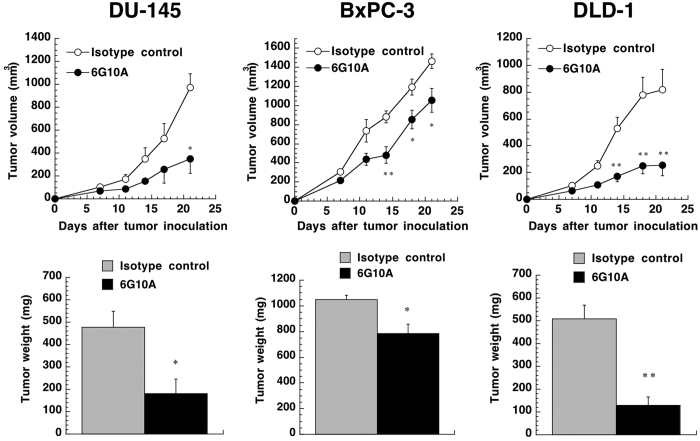
Effect of anti-CXADR antibody 6G10A on the growth of various subcutaneous tumors *in vivo*. DU-145, BxPC-3, or DLD-1 cells were injected subcutaneously into male nude mice. Antibodies (250 μg/day) were administered intravenously 1, 7, and 14 days after the cancer cell injection. Mice were sacrificed 21 days after the cancer cell injection, and the tumors were excised and weighed. The values are means ± SEM (n = 3). *P,0.05 and **P < 0.01 versus the control values.

When we examined the expression of CXADR in other cancer cell lines, we found that human pancreatic cancer cell line BxPC-3 and human colorectal cancer cell line DLD-1 both expressed CXADR, in addition to DU-145 cells (Fig. 1a). We then assessed the anti-tumor effect of anti-CXADR antibody 6G10A against xenograft models of these three cancer cell lines. As shown in Fig. 4, 6G10A significantly inhibited the growth of xenograft tumors of each of the CXADR-expressing cancer cell lines without any severe adverse effect on host mice (Supplementary Fig. 5).

**Figure 5:**
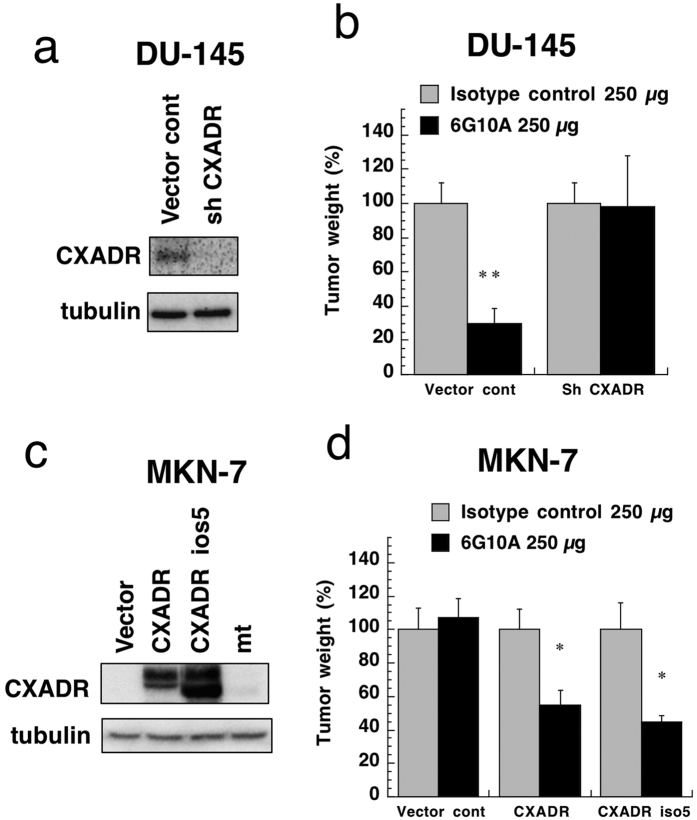
Effect of knockdown or overexpression of CXADR on 6G10A anti-tumor activity *in vivo*. (**a**) CXADR expression in DU-145 cells transfected with control vector or an shRNA expression vector. The gels have been run under the same experimental conditions and cropped to show protein bands corresponding to CXADR or tubulin as indicated. (**b**) DU-145 cells expressing a control vector (Vector cont) or CXADR shRNA vector (sh CXADR) cells were injected subcutaneously into male nude mice. Antibodies (250 μg/day) were administered intravenously 1, 7, and 14 days after the cancer cell injection. Mice were sacrificed 21 days after the cancer cell injection, and the tumors were excised and weighed. The values are means ± SEM (n = 3). **P < 0.01 versus the control values. (**c**) CXADR expression in MKN-7 cells expressing a control vector (Vector cont), CXADR, CXADR iso5, or mutant CXADR (mt). The gels have been run under the same experimental conditions and cropped to show protein bands corresponding to CXADR or tubulin as indicated. (**d**) MKN-7 cells expressing a control vector (Vector cont), CXADR, or CXADR iso5 were injected subcutaneously into female nude mice. Antibodies (250 μg/day) were administered intravenously 1, 7, and 14 days after the cancer cell injection. Mice were sacrificed 21 days after the cancer cell injection, and the tumors were excised and weighed. The values are means ± SEM (n = 5). **P < 0.01 versus the control values.

In order to confirm the physiological target of 6G10A, we constructed cancer cells with knockdown or overexpression of the CXADR gene. Expression of CXADR in DU-145 cancer cells was suppressed by transfection of a CXADR shRNA expression vector (Fig. 5a). When we assessed the anti-tumor effect of 6G10A *in vivo*, we found that it failed to inhibit the growth of DU-145 tumor cells that lacked CXADR expression (Fig. 5b). Although five isoforms of CXADR were reported^11^, PCR experiments suggested that the cancer cell lines used herein expressed isoforms 1 and 5, and an unexpected shorter gene (Supplementary Fig. 6). We cloned these genes and transfected expression vectors containing them into MKN-7 human gastric cancer cells, because MKN-7 cells basically did not express CXADR (Fig. 1a). Although the shorter gene was not expressed on the cell surface and was considered to be an artifact, isoform 1 (designated as CXADR) and isoform 5 (iso5) were successfully expressed on the surface of MKN-7 cells (Fig. 5c and Supplementary Fig. 7). We then examined the anti-tumor effect of 6G10A against these transfectants that overexpressed CXADR. Although it did not affect the growth of xenograft tumor of MKN-7 cells transfected with a control vector, 6G10A did inhibit the tumor growth of MKN-7 cells expressing CXADR and also CXADR iso5 (Fig. 5d).

### Anti-CXADR antibody treatment enhanced ADCC and CDC

**Figure 6:**
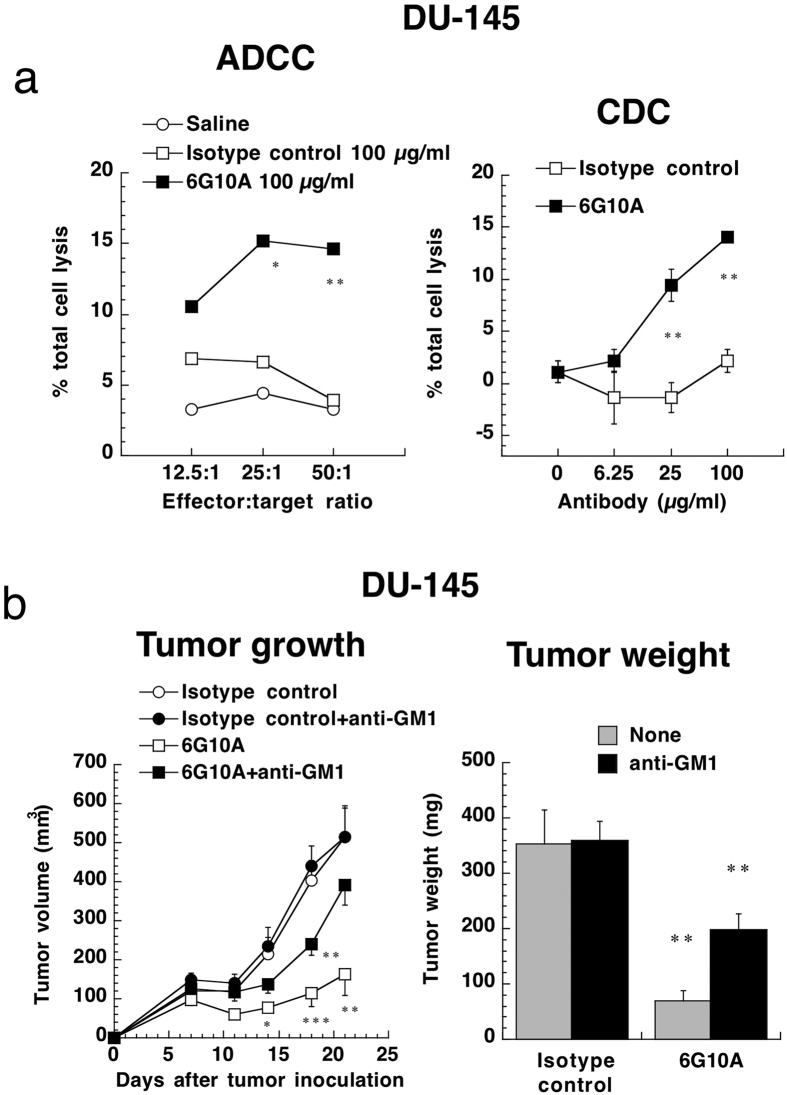
Effect of anti-CXADR antibody 6G10A on ADCC and CDC. **(a**) For ADCC activity, DU-145 cells were incubated with splenocytes from male nude mice in the presence of the indicated antibodies at 100 μg/ml for 4 h. For CDC activity, DU-145 cells were incubated with 10% rabbit complement in the presence of the indicated antibodies for 4 h. Cell lysis was determined using calcein AM. The values are means ± SEM (n = 3). *P < 0.05 and **P < 0.01 versus the control values. (**b**) DU-145 cells were injected subcutaneously into male nude mice. To deplete NK cells in the mice, anti-asialo GM1 antibodies (100 μg/day) were administered intravenously −1, 6, and 13 days after the cancer cell injection. Anti-CXADR antibody 6G10A (250 μg/day) was administered intravenously 1, 7, and 14 days after the cancer cell injection. The mice were sacrificed 21 days after the cancer cell injection, and the tumors were excised and weighed. The values are means ± SEM (n = 5). *P < 0.05 and **P < 0.01 versus the control values.

Since many antibodies exert their anti-tumor activities through antibody-dependent cellular cytotoxicity (ADCC) and complement-dependent cytotoxicity (CDC)^12^, we evaluated the ADCC and CDC activity of 6G10A. We labeled DU-145 cells using calcein AM as a target cell and used splenocytes derived from nude mice as a source of NK cells. As a result, we found that 6G10A significantly augmented ADCC activity against DU-145 cells (Fig. 6a). Moreover, NK cells were frequently found in DU-145 tumor tissues treated with 6G10A compared to isotype control-treated ones (Supplementary Fig. 8). 6G10A was also found to significantly enhance CDC activity against DU-145 cells when using rabbit complement (Fig. 6a). BALB/c nude mice have B-cells, NK cells and complement. To confirm these actions *in vivo*, we used anti-asialo GM1 antibody to deplete NK cells, because ADCC activity is exerted mainly by NK cells^12^. We found that about half of the ADCC anti-tumor activity against the DU-145 cells was reduced by anti-asialo GM1 antibody treatment (Fig. 6b and Supplementary Fig. 9), suggesting that 6G10A exerted anti-tumor activity through both ADCC and CDC activities.

### Expression of CXADR in human normal and tumor tissues

**Figure 7:**
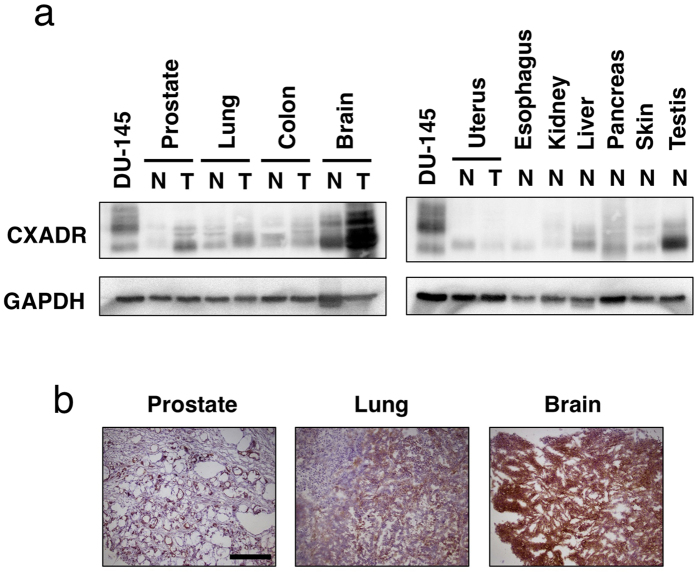
CXADR expression in human normal and tumor tissues. (**a**) CXADR expression in the indicated tissues was determined by Western blotting using lysates from various tissues. DU-145 cells were used as a positive control. N, normal; T, tumor. The gels have been run under the same experimental conditions and cropped to show protein bands corresponding to CXADR or GAPDH as indicated. (**b**) Frozen sections from the indicated tumor tissues (BioChain) were stained with anti-CXADR antibody 6G10A. Scale bar is 100 μm.

We examined the expression levels of CXADR protein in several normal and tumor tissues using a commercially available antibody against CXADR. We examined lysates of various tissues and found that CXADR was expressed in tumor tissues of prostate, lung, and brain at higher levels than in respective normal tissues (Fig. 7a). Differently mobilized bands of CXADR were detected, which is likely attributed to different levels of glycosylation and the presence of isoforms^11^. Some normal tissues, such as liver and pancreas, expressed CXADR at marginal levels compared to tumor tissues and cancer cells, but normal testis expressed it at levels comparable to cancer cells (Fig. 7a).

We next made biotinylated 6G10A and used it for staining frozen sections and found that it stained tumor tissues of prostate, lung, and brain (Fig. 7b). Furthermore, 6G10A reacted with normal tissues of skin, prostate, and kidney (Supplementary Fig. 10).

## Discussion

Almost all anti-tumor antibodies developed previously recognize cell surface or secreted proteins and act directly by neutralizing or indirectly through ADCC and CDC^12^. Since SST-REX can easily isolate proteins that contain a membrane-anchoring or secretion signal sequence, which can then be used as antigens for immunization^3^,^4^, we were able to identify several genes that correlated with high tumorigenicity. We chose CXADR as a target for the development of anti-tumor antibodies because CXADR has not been clearly linked to cancer^7^.

LNCaP-CR, a highly tumorigenic androgen receptor-positive subline of LNCaP cells^6^, expressed higher levels of CXADR than the parental LNCaP cells (Fig. 1a). While AR-negative DU-145 cells also expressed CXADR, PC-3, another AR-negative prostate cancer cell line, did not (Fig. 1a). Thus, it appears that CXADR expression did not correlate with AR expression and androgen sensitivity.

Saito *et al*. reported that CXADR suppresses anoikis of cancer cells and renders tumorigenicity^7^. In contrast, some reports have shown that the expression level of CXADR inversely correlates with the growth of colorectal and gastric cancer cell lines^8^,^9^. In this study, however, knockdown and overexpression of CXADR did not drastically suppress or increase the growth of cancer cells *in vitro* (Supplementary Fig. 11). Stecker *et al*. reported that knockdown of CXADR inhibits α-catenin expression^13^, but we could not obtain the same results in this study (data not shown). Thus, our results suggest that CXADR could not play a critical role in tumorigenesis. Instead CXADR could work as a neutral target for the antibody recognition. However, there are some reports showing the positive or negative involvement of CXADR in tumorigenesis as described above^7^,^8^,^9^. Since we could not exclude the possibility that the dependence of CXADR on cell growth or survival might differ among cell lines, further investigation is required to elucidate the precise role of CXADR on cancer cell growth.

Although the anti-CXADR antibody 6G10A did not directly affect the growth of LNCaP-CR cells, it apparently suppressed the growth of DU-145 cells (Supplementary Fig. 12). Furthermore, 6G10A exhibited anti-tumor activity against DU-145 cells, even in NOG mice in which T-cells, B-cells, NK cells and complement are almost entirely deficient (Supplementary Fig. 13). While main anti-tumor mechanisms of 6G10A are ADCC and CDC activities, we are not able to exclude the possibility that 6G10A could have a direct anti-proliferative activity against some cancer cells. However, 6G10A did not affect cell viability, migration, and apoptosis in cancer cells (Supplementary Figs 12 and 14) suggesting that 6G10A inhibited the cell proliferation. While the intracellular internalization is one of characteristics of antibodies, 6G10A was not incorporated intracellularly even after overnight incubation (Supplementary Fig. 15). We are now trying to reveal the precise mechanism of 6G10A action on the cell proliferation.

Regarding CXADR as a target for 6G10A, overexpression of CXADR isoform 1 as well as isoform 5 rendered MNK-7 cancer cells sensitive to 6G10A (Fig. 5d). Isoforms 1 and 5 of CXADR have the same extracellular domain (Supplementary Fig. 16), but isoform 5 has a shorter intracellular domain. Thus, it is clear that 6G10A recognizes the same region of isoforms 1 and 5. It is difficult to determine whether DU-145 cells express isoform 1, 5, or both, because these isoforms are nearly the same size and variously glycosylated (Fig. 5c)^11^. However, an shRNA expression vector against CXADR targeted and downregulated both isoforms 1 and 5 (Fig. 5a). We showed that 6G10A failed to exert anti-tumor activity against cancer cells when CXADR was downregulated (Fig. 5b). Thus, our results clearly showed that the anti-tumor activity of 6G10A depends on the expression of CXADR on cancer cell surface.

Our present results show that anti-CXADR antibody 6G10A exerts potent anti-tumor activity primarily through both ADCC and CDC activities. Others, in addition to us, have reported that CXADR is expressed in some normal tissues such as liver, pancreas, and testis (Fig. 7a)^8^,^9^,^14^. CXADR plays critical roles in the early embryonic development of heart and the lymphatic system^15^,^16^. Since it is still unknown what role CXADR plays in an adult body, we are now studying the action of our antibody against normal tissues in respect to predicting adverse effects. Our present results showed that 6G10A reacted with normal tissues of skin, prostate, and kidney (Supplementary Fig. 10). However, it reacted epithelium in prostate cancer adjacent normal tissues (Supplementary Fig. 17a,b), but it only reacted with myoepithelium in normal prostate (Supplementary Fig. 17c–e). Since 6G10A did not react with normal epithelium from which prostate cancer arose, there is a possibility that the cancer adjacent normal tissues could be affected by cancer cells. While 6G10A stained mainly renal tubules in kidney, it did not stain glomeruli (Supplementary Fig. 17f–i). Thus, 6G10A could not affect the glomerular filtration. In respect to skin, 6G10A reacted with basal cells in epidermis (Supplementary Fig. 17j–m). Although there is the possibility that 6G10A could affect such normal tissues, for conclusion we need further evaluation because of low numbers and qualities of frozen tissue sections. In contrast, our results suggest that CXADR is highly expressed in tumor tissues of prostate, lung, and brain (Fig. 7a). Others have reported high expression of CXADR in tumor tissues, such as neuroendocrine lung cancer^17^ and endometrial adenocarcinoma^18^. We intend to evaluate CXADR expression in various tumor tissues in a future study.

Taken together, these results have clearly shown that our anti-CXADR antibody 6G10A has great potential as an anti-tumor drug. We are now trying to develop a human-mouse chimeric 6G10A to create a new anti-tumor antibody for cancer immunotherapy.

## Materials and Methods

### Cell lines and reagents

Human prostate cancer LNCaP cells were obtained from DS Pharma, and its highly tumorigenic subline LNCaP-CR was established in our laboratory^6^. Human prostate cancer DU-145 cells, human colorectal cancer DLD-1 cells, and human pancreatic cancer BxPC-3 cells were obtained from the American Type Culture Collection (ATCC). Human prostate cancer PC-3 cells were purchased from DS Pharma. Green fluorescence protein (GFP)-expressing LNCaP-CR cells and human gastric cancer MKN-7 cells were prepared as described^19^. All cancer cell lines were maintained in Dulbecco’s Modified Eagle’s Medium (DMEM) (Nissui) supplemented with 10% fetal bovine serum (FBS; Sigma), 100 units/ml penicillin G (Invitrogen), and 100 μg/ml streptomycin (Invitrogen) at 37 °C with 5% CO_2_. Ba/F3, a murine IL-3 dependent pro-B cell line, was maintained in RPMI-1640 medium supplemented with antibiotics described above and 10% FBS.

Anti-CXADR (HPA003342) and anti-tubulin antibodies were purchased from Sigma. Anti-CXADR (H-300) was from Santa Cruz Biotechnology. Mouse IgG2a isotype control antibodies were obtained from Sigma and Cell Lab, and mouse IgG2b isotype control antibodies were from Cell Lab. Calcein AM was from Molecular Probes and Low-Tox-M rabbit complement was from Cedarlane. Anti-asialo GM1 antibody was from Wako Pure Chemical Industries, Ltd.

### SST-REX method

The SST-REX method was performed as described^3^,^4^. Total RNA was purified from LNCaP-CR and LNCaP cells using Trizol (Invitrogen) and mRNA was isolated using Fast-Track 2.0 kits (Invitrogen). cDNA was synthesized using random hexamers with SuperScript Choice System (Invitrogen) according to the manufacturer’s instructions and inserted into *Bst*XI sites of pMX-SST vector^3^,^4^ using *Bst*XI adaptors (Invitrogen). The ligated vectors were electroporated into *E. coli* using an *E-coli* Pulser (Bio-Rad) at 1.8 kV. Then, the cDNA library was prepared by culturing the transfected *E. coli.* High-titer retroviruses from the above cDNA library were produced using the packaging cell line Plat-E (Cell Biolabs). Ba/F3 cells were infected with the retroviruses using Polybrene (Chemicon). Ba/F3 clones that grew in the absence of IL-3 were selected. Genomic DNA extracted from the IL-3-independent Ba/F3 clones was applied to PCR to recover the integrated cDNAs using the PCR primers, 5′-TAATACGACTCACTATAGGGCGCGCAGCTGTAAACGGTAG-3′ and 5′-ATTAACCCTCACTAAAGGGAGGGGGTGGACCATCCTCTA-3′. The PCR products were sequenced using BigDye Terminator v3.1 Cycle Sequencing kits with 5′-ATTAACCCTCACTAAAGGGAGGGGGTGGACCATCCTCTA-3′ as a primer.

### Development of anti-CXADR antibodies

Four-week-old female BALB/c mice were used for immunization. One day before the first immunization, the mice were injected subcutaneously with TiterMax Gold (Alexis Biochemicals). Then, Ba/F3 cells expressing human CXADR were injected intraperitoneally into the mice every two days for a total of four times. Inguinal and popliteal lymph nodes from immunized mice were isolated and fused with myeloma P3U1 cells. The resulting hybridomas were cultured in DMEM containing 15% FBS, 100 μM hyposiantine, 0.4 μM aminopterin, 16 μM thymidine (HAT; Sigma), and 50 pg/ml of murine IL-6 for 2 weeks. Conditioned medium of the hybridoma clones was checked for reactivity to CXADR by FACS analysis. Clones that produced anti-CXADR antibodies were grown in Hybridoma SFM II medium (Invitrogen) at 37 °C with 5% CO_2_. Conditioned medium of the selected clones was applied to protein-A sepharose columns (GE Healthcare) and bound antibodies were eluted with 1 M arginine (pH 4.0). Antibodies were dialyzed against PBS and used as purified antibodies.

### Determination of epitopes of antibodies

Ba/F3 cells expressing various deletion mutants of CXADR were constructed. From the N-terminus, 83, 133, 181, 230, and 237 amino acid lengths of the CXADR gene were synthesized by PCR using the following primers: general forward primer, 5′-CCGGAATTCCCACGGCACGGCAGCCACCATGG-3′; 83 reverse primer, 5′-TTTTCCTTTTGCGGCCGCGTAGTCATCATAAATTTTGTCTCC-3′; 133 reverse primer, 5′-TTTTCCTTTTGCGGCCGCAATCTTCTTATTTGCAACACCAGG-3′; 181 reverse primer, 5′-TTTTCCTTTTGCGGCCGCTGAGTCAGACAATTTTTGCCACTC-3′; 230 reverse primer, 5′-TTTTCCTTTTGCGGCCGCGGACAACGTTTAGACGCAACAG-3′; 237 reverse primer, 5′-TTTTCCTTTTGCGGCCGCTCCAGCTTTATTTGAAGGAGGGAC-3′. The PCR products were digested with *Eco*RI and *Not*I and inserted into the pMX-SST vector. Then, Ba/F3 cells expressing the CXADR mutants were established as described above. Using these cells, we examined reactivity of anti-CXADR antibodies against various antigens of CXADR by FACS analysis and determined the epitopes of the anti-CXADR antibodies (Supplementary Fig. 18). As a result, anti-CXADR antibodies described in this study were found to recognize the specific regions of CXADR, as shown in Fig. 1c.

### Knockdown of CXADR

The SureSilencing shRNA plasmids for human CXADR (NM_001338) and negative control plasmids were purchased from QIAGEN (Hilden, Germany). Each plasmid carries an shRNA under the U1 promoter with puromycin resistance gene as a marker. The shRNA sequences are as follows: CXADR shRNA#1, 5′-AAAGGAAGTTCATCACGATAT-3′; CXADR shRNA#2, 5′-GAAGGTGGATCAAGTGATTAT-3′; negative control shRNA, 5′-GGAATCTCATTCGATGCATAC-3′. Transfection of DU-145 cells with the SureSilencing shRNA plasmids was performed with FuGENE HD transfection reagent (Promega) according to the manufacturer’s instructions. Stable shRNA-expressing cells were established after 20 days of Puromycin (0.5 μg/ml) selection.

### Overexpression of CXADR

Total RNA was isolated from LNCaP, DU-145, and DLD-1 cells using RNeasy Minikits (Qiagen). cDNAs were synthesized using AMV reverse transcriptase (Promega) and CXADR isoforms of PCR products were amplified using the following primers: general sense primer, 5′-ATGGCGCTCCTGCTGTGCTTCGTG-3′; isoform 1 revers primer, 5′-CTATACTATAGACCCATCCTTGC-3′; isoform 2 and 3 reverse primer, 5′-CTACCTTAGCAGGTGGGAGAGTC-3′; isoform 4 reverse primer, 5′-TTACTGCCGATGTAGCTTCTGGC-3′; isoform 5 reverse primer, 5′-TTATACAACTGTAATTCCATCAG-3′. Full-length human CXADR cDNA fragments of isoform 1 and 5 containing *Nhe*I and *Xho*I sites were generated by PCR using DU-145 cDNA, PfxDNA polymerase (Invitrogen) and the following primers: general sense primer, 5′-TATGCTAGCCACCATGGCGCTCCTG-3′; isoform 1 reverse primer, 5′-TATCTCGAGTACTATAGACCCATCCTTGCT-3′; isoform 5 reverse primer, 5′-TATCTCGAGTACAACTGTAATTCCATC-3′. The *Nhe*I/*Xho*I fragments of CXADR isoforms were inserted into an NBEX-Flag vector. The NBEX-Flag vector was constructed by insertion of the C-terminal Flag-tag sequence into pcDNA3.1/Hygro(+) cleaved with *Nhe*I and *Pme*I. The sequence of all expression vectors was confirmed by sequencing. MKN-7 cells were transfected with the expression vectors using the Lipofectamine reagent and selected with hygromycin.

### Western blot

Cell lysates were prepared and applied to Western blots as described before^20^. Lysates of normal and tumor tissues from various human organs were obtained from BioChain.

### FACS analysis

Cells were resuspended in PBS containing 0.1% sodium azide. The cells were incubated with anti-CXADR antibodies for 30 min and then with anti-mouse PE antibodies. After washing with PBS, the cells were analyzed by FC500 MPL (Beckman Coulter).

### Immunofluorescence

Cells were cultured on glass cover slips in 6-well plates at 1 × 10^5^ cells/well in DMEM supplemented with 10% FBS for two days. The cells were washed with PBS and fixed with 4% formaldehyde in PBS for 15 min. The fixed cells were stained with anti-CXADR antibodies and then with anti-mouse IgG2a Alexa Fluor 546. The stained cells were analyzed with a Leica DM IRB fluorescence microscope.

### Cell growth

Cells were inoculated into 96-well plates at 5 × 10^3^ cells/well in 0.1 ml of DMEM supplemented with 10% FBS and cultured for three days with the indicated concentrations of antibodies. Cell growth was determined using 3-(4,5-dimethyllythiazol-2-yl)-2,5-diphenyltetrazolium bromide (MTT; Sigma) as described^21^. For cell viability, the cells were trypsinized and stained with 0.4% trypan blue. In case of wound healing assays, the cells were grown to confluent. After culturing in reduced 1% dialyzed FBS overnight, the confluent cells were scratched by plastic pipette tips (0 h) and further cultured in the presence of antibodies for 24 h.

### Anti-tumor activity *in vivo*

Animal experiments were approved by the Institutional Committee for Animal Experiments in the Institute of Microbial Chemistry and performed in accordance with the relevant guidelines and regulations to minimize animal suffering. Male or female BALB/c nude mice, six weeks old, were purchased from Charles River Breeding Laboratories; female NOG mice were obtained from the Central Institute of Experimental Animals (Kawasaki, Japan) and maintained in a specific pathogen-free barrier facility according to our institutional guidelines. Mice were used for experiments at eight weeks of age. Cancer cells (8 × 10^6^) were trypsinized and resuspended in 0.3 ml of 10% FBS-DMEM and then combined with 0.5 ml of growth factor-reduced Matrigel (BD Biosciences). 100 μl of the cell suspension (1 × 10^6^ cells) was injected subcutaneously in the left flank of the mice. Antibodies were administered intravenously at the indicated days and concentrations. Tumor volume was estimated using the following formula: tumor volume (mm^3^) = (length × width^2^)/2. Tumors were surgically dissected on the indicated days.

For orthotopic implantation of prostate cancer cells, male mice were anesthetized by intraperitoneal injection of pentobarbital sodium at 66.7 mg/kg. After making a small median abdominal incision, cancer cells (1 × 10^6^) in 20 μl of the Matrigel-containing medium were inoculated into the murine prostate lobe using a 30-gauge needle (Nipro). The abdominal wall was sutured and the skin was closed with an AUTOCLIP applier (Becton-Dickinson). Mice were euthanized on the indicated days after the tumor inoculation.

### ADCC and CDC activity

ADCC activity was assessed as described^22^. Spleens were removed aseptically from nude mice and single cell suspensions were obtained by dispersing the spleens using a syringe and pressing through stainless steel mesh. Erythrocytes were lysed by a 10 s-exposure to ice-cold distilled water. Splenocytes were washed with RPMI1640 and resuspended in 10% FBS RPMI1640 as effector cells. DU-145 cells (5 × 10^5^ cells/ml) were labeled with 10 μg/ml calcein AM in serum-free RPMI1640 for 30 min at 37 °C, washed three times with 10% FBS RPMI1640 (complete medium), and further incubated in complete medium for 1 h at 37 °C. After washing three times with complete medium, the labeled DU-145 cells (5 × 10^4^ cells) were mixed with the effector cells at the indicated ratios and further incubated for 4 h at 37 °C. After centrifugation, cell-free supernatants were carefully removed and fluorescence intensity was measured using an excitation at 485 nm and emission at 538 nm. Cytolytic activity (as % lysis) was calculated using the following formula: % of specific lysis = (E - S)/(M - S) × 100 (where E is the fluorescence released in experimental cultures of target cells and effector cells; S is the spontaneous fluorescence released in cultures containing only target cells; M is the maximum fluorescence obtained by adding lysis buffer containing 0.5% Triton X-100, 10 mM Tris-HCl (pH 7.4), and 10 mM EDTA to target cells to lyse all cells).

For CDC activity, DU-145 cells were labeled with calcein AM as described above and inoculated into 96-well plates at 5 × 10^4^ cells/well in 0.1 ml of RPMI1640 medium supplemented with 10% FBS. The cells were pre-incubated with the indicated concentrations of antibodies for 1 h at 37 °C and then further incubated with the indicated concentrations of rabbit complement for 4 h at 37 °C. Fluorescence intensity in cell-free supernatants was measured as described above.

### Immunohistochemistry

Frozen normal and tumor tissue arrays were obtained from BioChain. The tissue arrays were fixed in ice-cold acetone for 10 min and dipped in 1% H_2_O_2_ in PBS for 5 min. Immunohistochemical staining was conducted with biotinylated anti-CXADR 6G10A antibody. The biotinylation of 6G10A antibody has been done using NHS-LC-Biotin (Thermo Scientific). The estimated visual intensity of 6G10A immunostaining was graded on arbitrary 4 point scales: negative (−), weakly positive (+), positive (++), and strong positive (+++). Paraffin-embedded xenograft tumors of DU-145 cells treated with 6G10A or isotype control antibodies were stained with anti-NCR1 antibody.

### Statistical analysis

All data are representative of at least three independent experiments with similar results. Statistical analyses were carried out using Student’s *t*-test.

## Additional Information

**How to cite this article:** Kawada, M. *et al*. A novel monoclonal antibody targeting coxsackie virus and adenovirus receptor inhibits tumor growth *in vivo. Sci. Rep.* **7**, 40400; doi: 10.1038/srep40400 (2017).

**Publisher's note:** Springer Nature remains neutral with regard to jurisdictional claims in published maps and institutional affiliations.

## Supplementary Material

Supplementary Information
