# Supplementary material for: A novel monoclonal antibody targeting coxsackie virus and adenovirus receptor inhibits tumor growth in vivo
Source: Sci Rep. 2017 Jan 11;7:40400. doi: 10.1038/srep40400 (PMC5225458; doi:10.1038/srep40400)
Supplement: Supplementary Information [file srep40400-s1.pdf]

Supplementary Information

A novel monoclonal antibody targeting coxsackie virus and adenovirus receptor inhibits tumor growth *in vivo*

Manabu Kawada[1,2], Hiroyuki Inoue[1], Masunori Kajikawa[3], Masahito Sugiura[3], Shuichi Sakamoto[1], Sakiko Urano[3], Chigusa Karasawa[3], Ihomi Usami[1], Mitsuru Futakuchi[4] & Tohru Masuda[1]

[1]Institute of Microbial Chemistry (BIKAKEN), Numazu, Microbial Chemistry Research Foundation, 18-24 Miyamoto, Numazu-shi, Shizuoka 410-0301, Japan
[2]Institute of Microbial Chemistry (BIKAKEN), Laboratory of Oncology, Microbial Chemistry Research Foundation, 3-14-23 Kamiosaki, Shinagawa-ku, Tokyo 141-0021, Japan
[3]Medical & Biological Laboratories Co., Ltd., 4-5-3 Sakae, Naka-ku, Nagoya, Aichi 460-0008, Japan
[4]Department of Molecular Toxicology, Nagoya City University Graduate School of Medical Sciences, 1 Kawasumi, Mizuho-cho, Mizuho-ku, Nagoya 467-8601, Japan

Correspondence and requests for materials should be addressed to M.Kaw. (email: kawadam@bikaken.or.jp)

Supplementary Figure 1

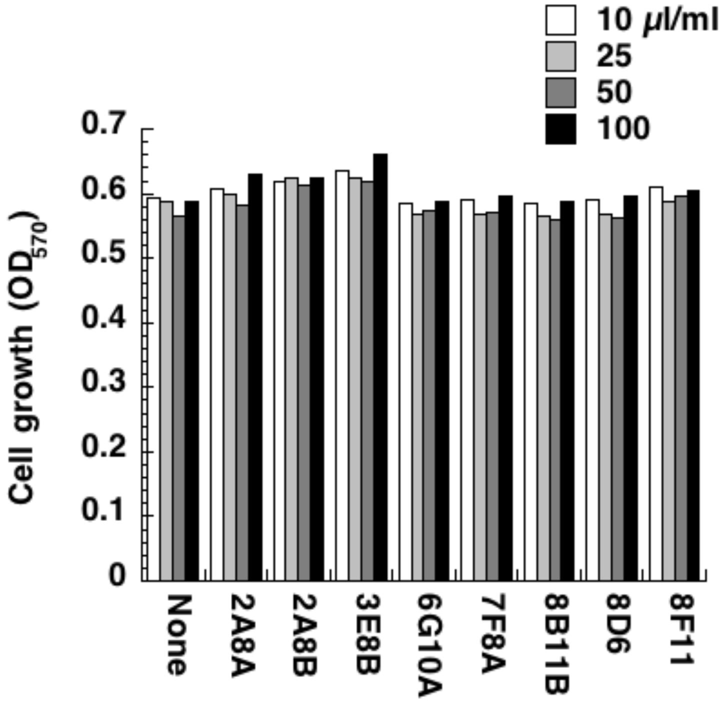

Supplementary Figure 1. Effect of anti-CXADR antibodies on the growth of LNCaP-CR cells *in vitro*. LNCaP-CR cells were cultured with the indicated concentrations of antibodies for 3 days. Cell growth was determined using MTT. The values are means of duplicate experiments. Each SE is less than 10%.

Supplementary Figure 2

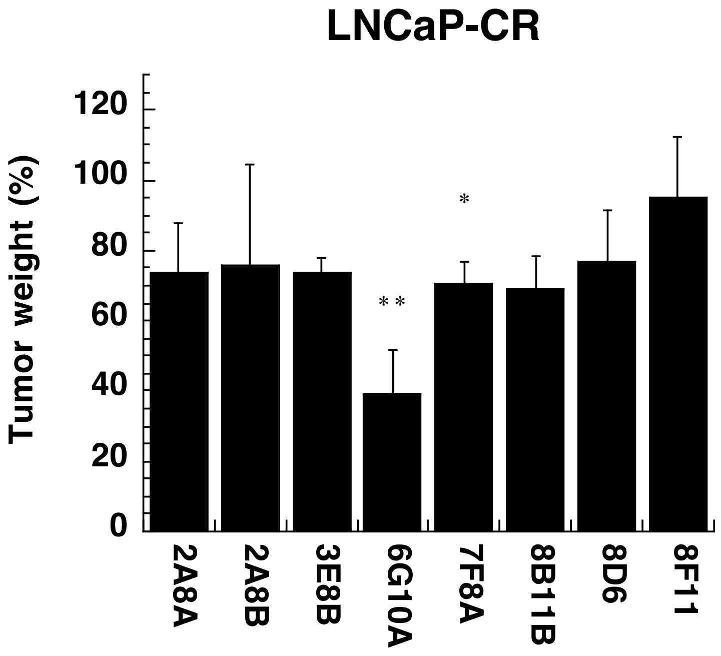

Supplementary Figure 2. Effect of anti-CXADR antibodies on the growth of LNCaP-CR tumors *in vivo*. LNCaP-CR cells were injected subcutaneously into male nude mice. One day after the cancer cell injection, the indicated antibodies were administered intravenously everyday for 11 days at 100 μl/day. Mice were sacrificed 21 days after the cancer cell injection, and the LNCaP-CR tumors were excised and weighed. The values are means ± SEM of percentage of control (n = 5). *P<0.05 and **P<0.01 versus the control values.

Supplementary Figure 3

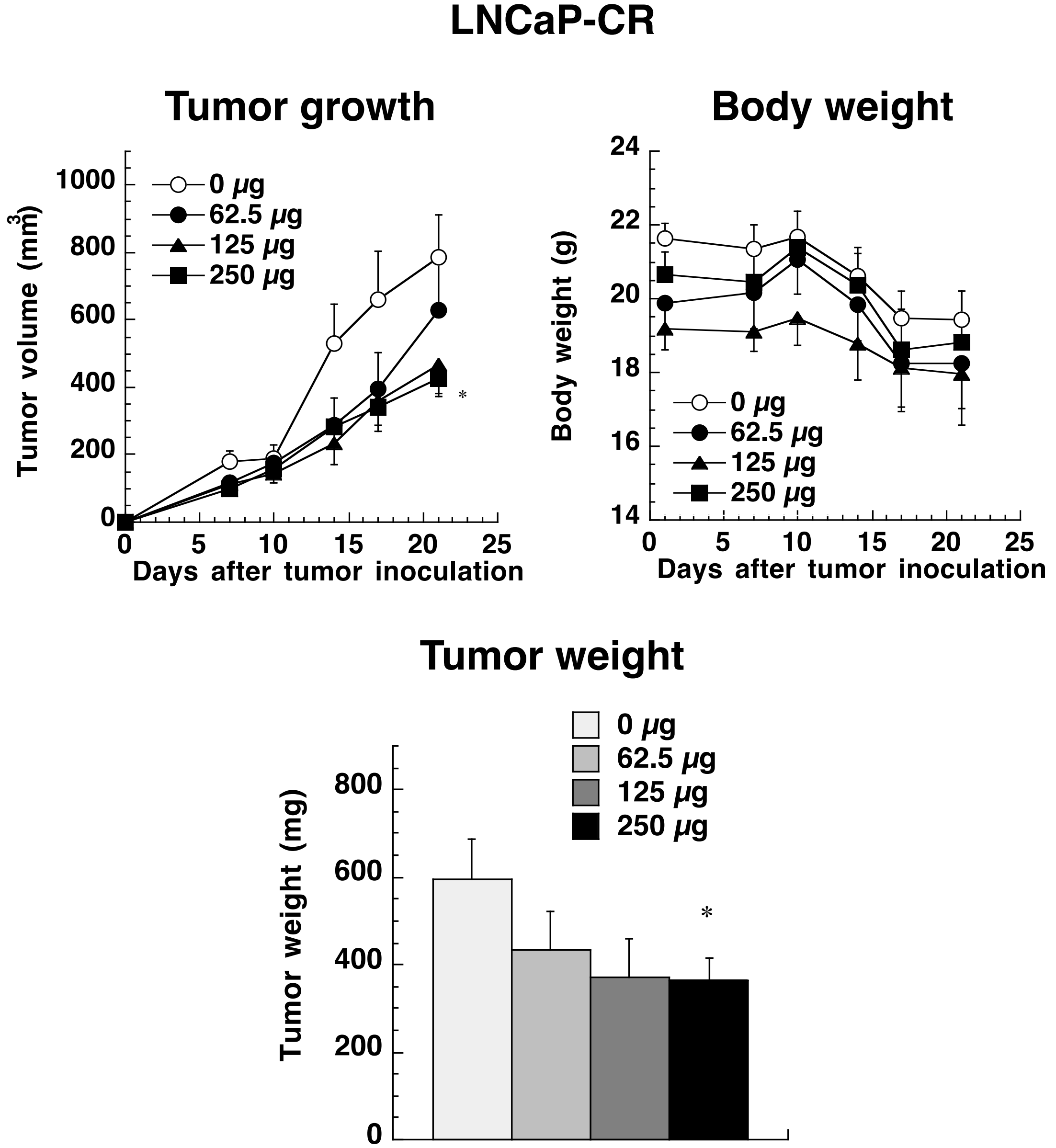

Supplementary Figure 3. Effect of anti-CXADR antibody 6G10A on the growth of LNCaP-CR subcutaneous tumors *in vivo*. LNCaP-CR cells were injected subcutaneously into male nude mice. The indicated doses of antibodies were administered intravenously 1, 7, and 14 days after the cancer cell injection. Mice were sacrificed 21 days after the cancer cell injection, and the LNCaP-CR tumors were excised and weighed. The values are means ± SEM (n = 5). *P<0.05 versus the control values.

Supplementary Figure 4

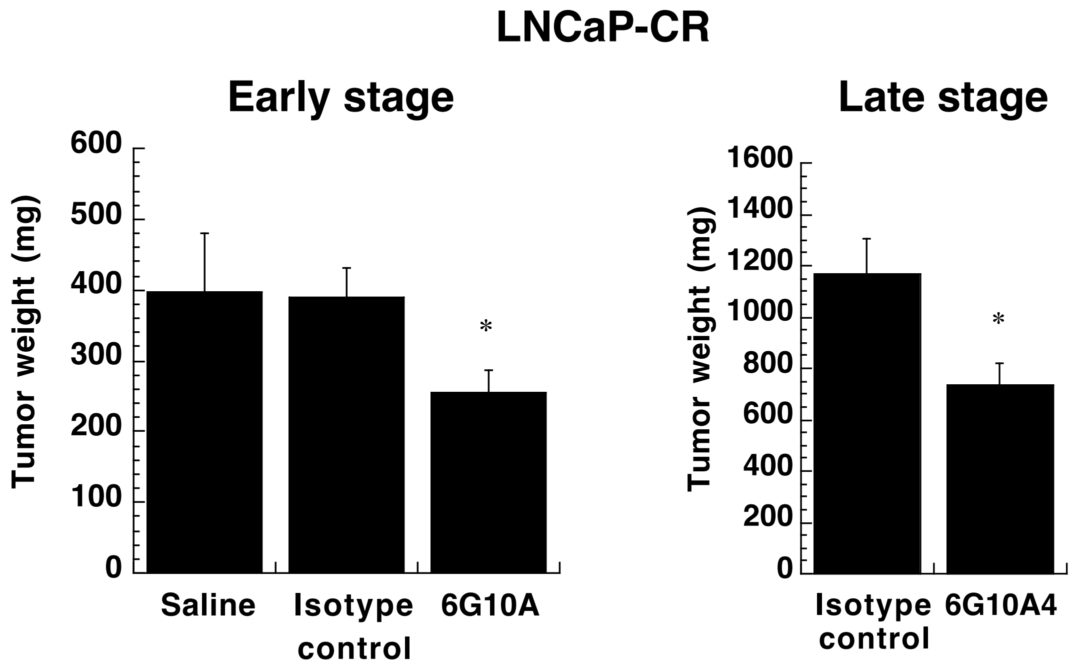

Supplementary Figure 4. Effect of anti-CXADR antibody 6G10A on the growth of late stage LNCaP-CR tumors *in vivo*. LNCaP-CR cells were injected subcutaneously into male nude mice. Antibodies (250 μg/day) were administered intravenously 1, 7, and 14 days (for early stage), or 14, 21, and 28 days (for late stage) after the cancer cell injection. Mice were sacrificed 21 (for early stage) or 35 (for late stage) days after the cancer cell injection, and the LNCaP-CR tumors were excised and weighed. The values are means ± SEM (n = 5). *P<0.05 versus the control values.

Supplementary Figure 5

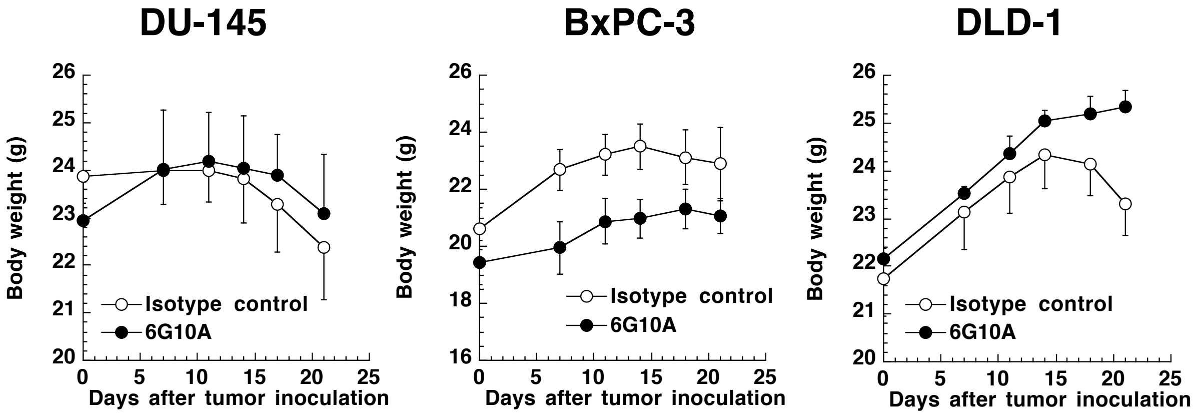

Supplementary Figure 5. Effect of anti-CXADR antibody 6G10A on mice body weight bearing various subcutaneous tumors *in vivo*. DU-145, BxPC-3, or DLD-1 cells were injected subcutaneously into male nude mice. Antibodies (250 μg/day) were administered intravenously 1, 7, and 14 days after the cancer cell injection. The values are means ± SEM (n = 3). This result is corresponding to Fig. 4.

Supplementary Figure 6

	isofrm				
primers	1	2	3	4	5
1	1098bp	-	-	-	-
2	~1020	759	603	-	~1020
3	~890	~625	~470	270	~890
4	-	-	-	-	1059

LNCaP DU-145 DLD-1

primers M 1 2 3 4 1 2 3 4 1 2 3 4

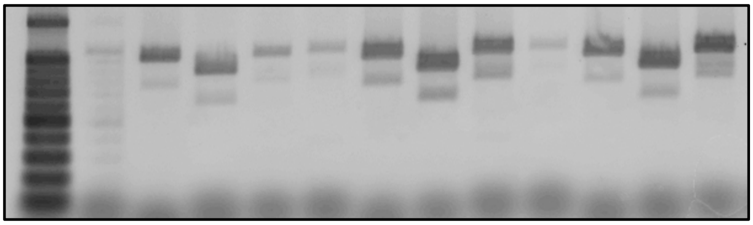

PCR

Supplementary Figure 6. Expression of CXADR isoforms in various cancer cell lines. PCR products for CXADR isoforms (1-5) were amplified using specific primer sets. Predicted sizes for each isoform are shown. M, 100bp DNA ladder maker.

Supplementary Figure 7

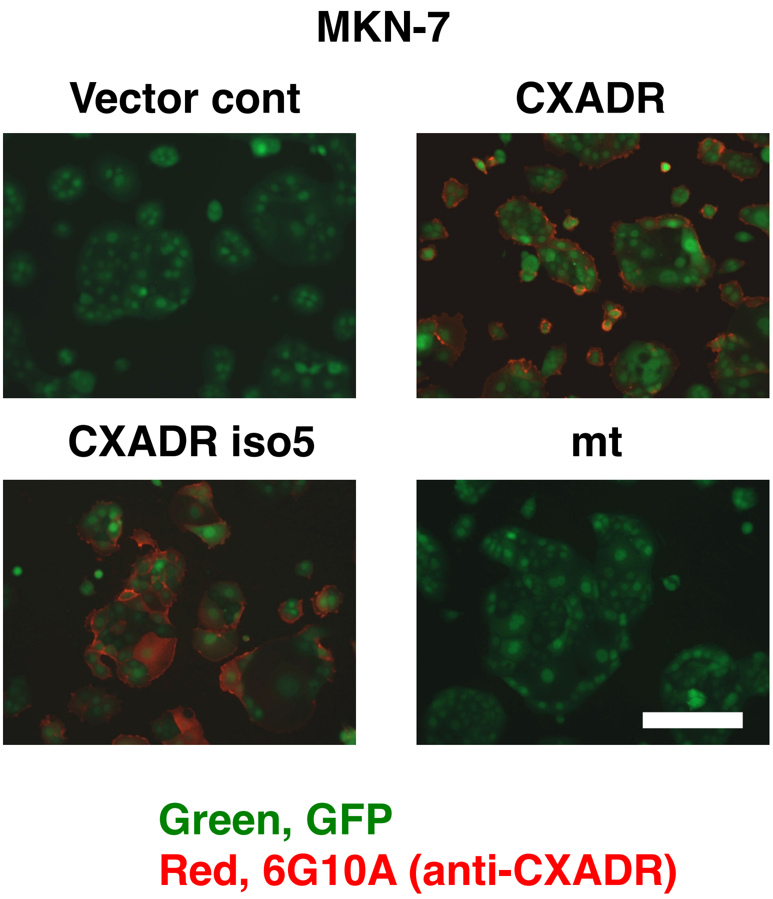

Supplementary Figure 7. Expression of CXADR isoforms in MKN-7 cells transfected with CXADR expression vectors. MKN-7 cells expressing a control vector (Vector cont), CXADR, or CXADR iso5 were fixed, stained with anti-CXADR antibody 6G10A, and analyzed by fluorescence microscopy. Scale bar is 200 μm.

Supplementary Figure 8

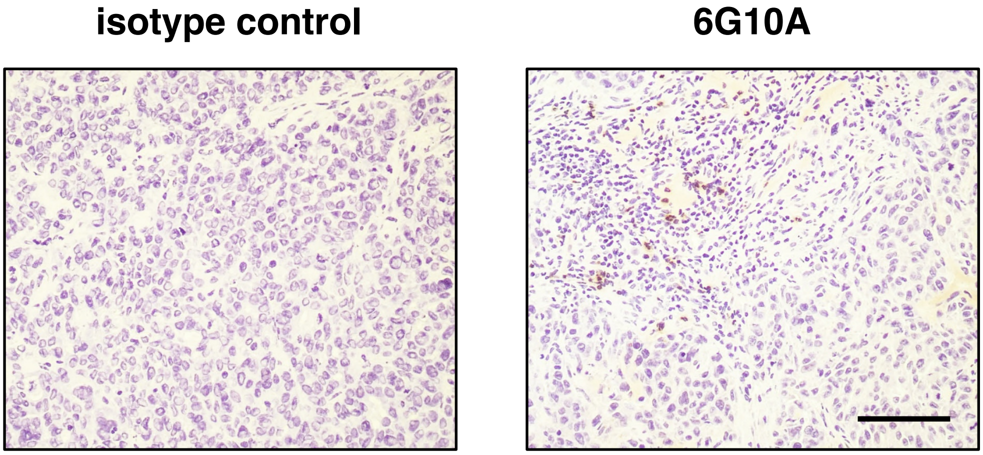

Supplementary Figure 8. Immunohistochemical analysis of NK cells in xenograft tumors of DU-145 cells. DU-145 cells were injected subcutaneously into male nude mice. Anti-CXADR antibody 6G10A clone (250 μg/day) was administered intravenously 1, 7, and 14 days after the cancer cell injection. Mice were sacrificed 21 days after the cancer cell injection and the DU-145 tumors were resected. Paraffin-embedded sections of the tumors were stained with anti-NCR1 antibody for the detection of NK cells. Scale bar is 100 μm.

Supplementary Figure 9

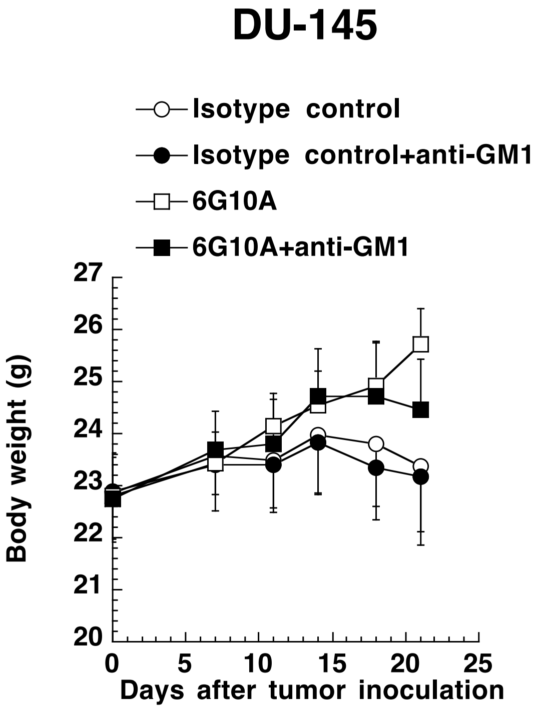

Supplementary Figure 9. Effect of anti-CXADR antibody 6G10A on mice body weight treated with anti-asialo GM1 antibodies *in vivo*. DU-145 cells were injected subcutaneously into male nude mice. NK cells were depleted with anti-asialo GM1 antibodies (100 μg/day) administered intravenously -1, 6, and 13 days after the cancer cell injection. Anti-CXADR antibody 6G10A clone (250 μg/day) was administered intravenously 1, 7, and 14 days after the cancer cell injection. The values are means ± SEM (n = 5). This result is corresponding to Fig. 6b.

Supplementary Figure 10

Tissue		Intensity
Breast	Normal	+
	Tumor	++
Brain	Normal	–
	Tumor	+++
Lung	Normal	–
	Tumor	++
Skin	Normal	++
	Tumor	+++
Prostate	Normal	++
	Tumor	+
Uterus	Normal	–
	Tumor	+++
Ovary	Normal	–
	Tumor	++
Kidney	Normal	+++
	Tumor	+++

Supplementary Figure 10. Immunoreactivity of anti-CXADR antibody 6G10A on human tissues. Frozen sections from the indicated normal and tumor tissues (n=1) (BioChain) were stained with biotinylated anti-CXADR antibody 6G10A. The estimated visual intensity of 6G10A immunostaining was graded on arbitrary 4 point scales: negative (-), weakly positive (+), positive (++), and strong positive (+++).

Supplementary Figure 11

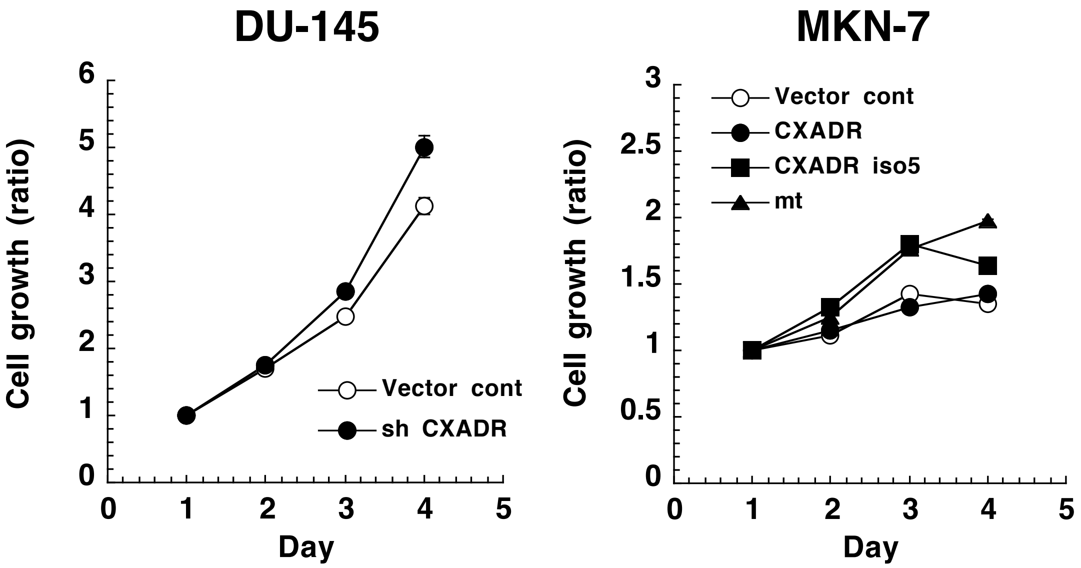

Supplementary Figure 11. Effect of knockdown or overexpression of CXADR on the growth of cancer cells *in vitro*. DU-145 cells expressing a control vector (Vector cont) or shRNA vector for CXADR (Sh CXADR) (left) or MKN-7 cells expressing a control vector (Vector cont), CXADR, CXADR iso5, or mutant CXADR (mt) (right) were cultured for the indicated days. Cell growth was determined using MTT. The values are means means ± SEM (n = 4).

Supplementary Figure 12

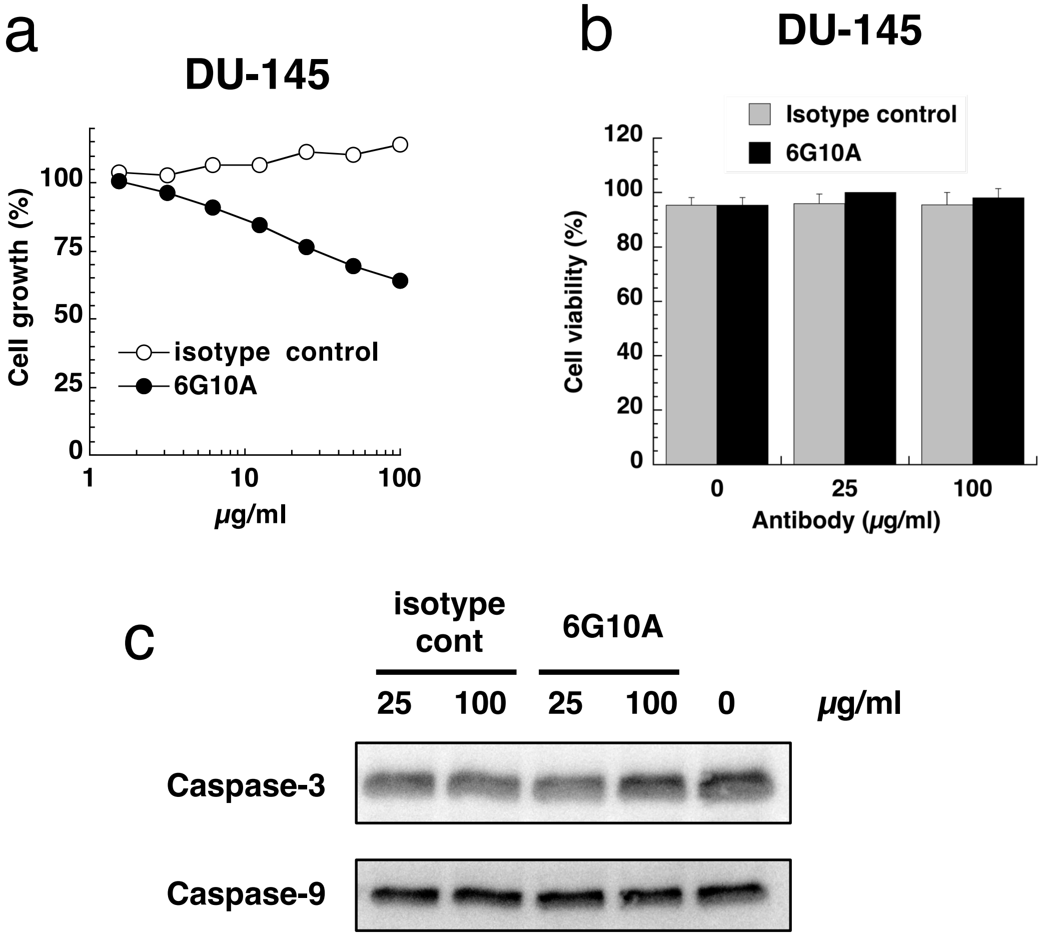

Supplementary Figure 12. Effect of anti-CXADR antibody 6G10A on the growth of DU-145 cells *in vitro*. (**a**) DU-145 cells were cultured with the indicated concentrations of antibodies for 3 days. Cell growth was determined using MTT. The values are means of duplicate experiments. Each SE is less than 10%. (**b**) DU-145 cells were cultured with the indicated concentrations of antibodies for 2 days. Cell viability was determined using trypan blue. The values are means ± SEM (n = 3). (**c**) DU-145 cells were cultured with the indicated concentrations of antibodies for 2 days. Expression of caspase-3 and caspase-9 was detected by Western blotting. The gels have been run under the same experimental conditions and cropped to show protein bands corresponding to caspase-3 or caspase-9 as indicated.

Supplementary Figure 13

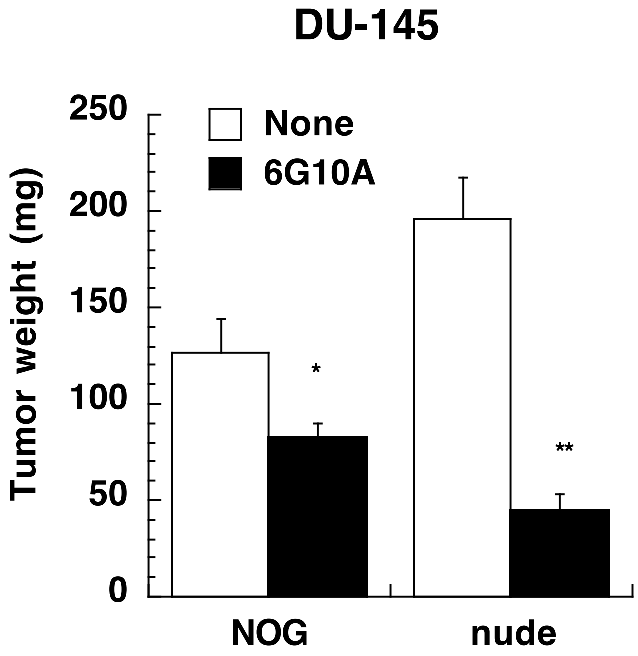

Supplementary Figure 13. Anti-tumor effect of anti-CXADR antibody 6G10A on NOG mice *in vivo*. DU-145 cells were injected subcutaneously into female NOG and female nude mice. Antibodies (250 µg/day) were administered intravenously 1, 7, and 14 days after the cancer cell injection. Mice were sacrificed 21 days after the cancer cell injection, and the tumors were excised and weighed. The values are means ± SEM (n = 5). *P<0.05 and **P<0.01 versus the control values.

Supplementary Figure 14

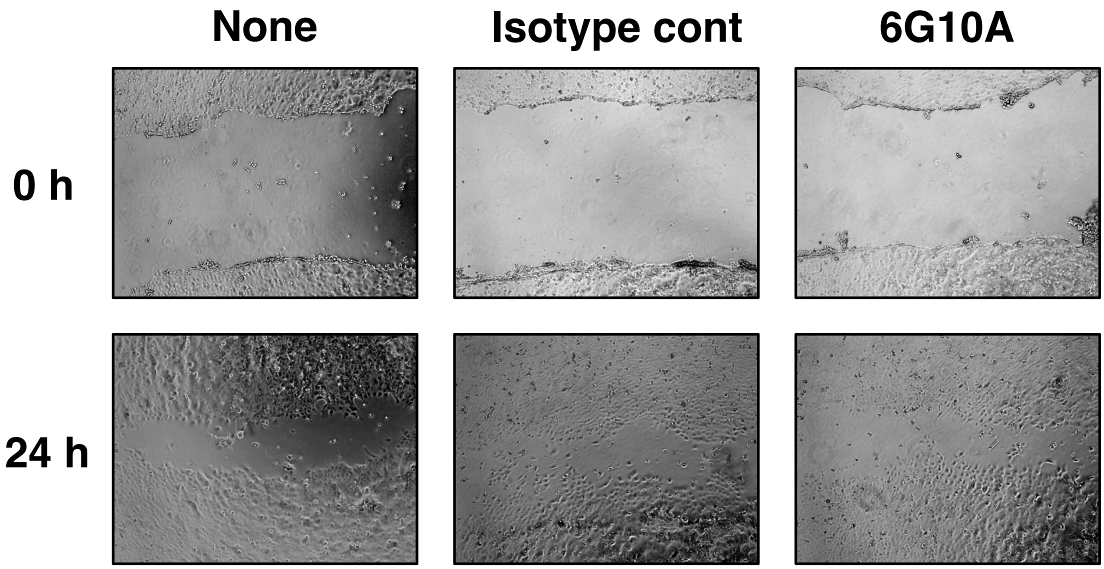

Supplementary Figure 14. Effect of anti-CXADR antibody 6G10A on wound healing assay of DU-145 cells *in vitro*. Confluent DU-145 cells were scratched by plastic pipette tips (0 h) and cultured with 100 μg/ml of the indicated antibodies for 24 h.

Supplementary Figure 15

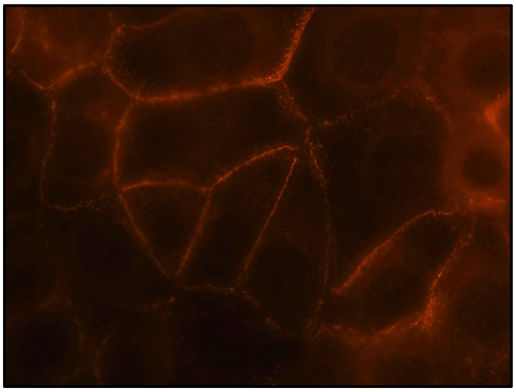

Supplementary Figure 15. Localization of anti-CXADR antibody 6G10A in DU-145 cells *in vitro*. DU-145 cells were treated with 5 μg/ml of 6G10A overnight. The cells were fixed with methanol and stained with anti-mouse IgG Alexa Fluor 546.

Supplementary Figure 16

```
File1: isoform5 protein
      1 -     353
File2: isoform1 protein
      1 -     366

Matching Percentage (Total Window: 93%, Alignment Window: 93%)

CXADR iso5   1 MALLLCFVLLCGVVDFARSLSITTPEEMIEKAKGETAYLPCKFTLSPEDQ      50
               ||||||||||||||||||||||||||||||||||||||||||||||||||
     CXADR   1 MALLLCFVLLCGVVDFARSLSITTPEEMIEKAKGETAYLPCKFTLSPEDQ      50

            51 GPLDIEWLISPADNQKVDQVIILYSGDKIYDDYYPDLKGRVHFTSNDLKS     100
               ||||||||||||||||||||||||||||||||||||||||||||||||||
            51 GPLDIEWLISPADNQKVDQVIILYSGDKIYDDYYPDLKGRVHFTSNDLKS     100

           101 GDASINVTNLQLSDIGTYQCKVKKAPGVANKKIHLVVLVKPSGARCYVDG     150
               ||||||||||||||||||||||||||||||||||||||||||||||||||
           101 GDASINVTNLQLSDIGTYQCKVKKAPGVANKKIHLVVLVKPSGARCYVDG     150

           151 SEEIGSDFKIKCEPKEGSLPLQYEWQKLSDSQKMPTSWLAEMTSSVISVK     200
               ||||||||||||||||||||||||||||||||||||||||||||||||||
           151 SEEIGSDFKIKCEPKEGSLPLQYEWQKLSDSQKMPTSWLAEMTSSVISVK     200

           201 NASSEYSGTYSCTVRNRVGSDQCLLRLNVVPPSNKAGLIAGAIIGTLLAL     250
               ||||||||||||||||||||||||||||||||||||||||||||||||||
           201 NASSEYSGTYSCTVRNRVGSDQCLLRLNVVPPSNKAGLIAGAIIGTLLAL     250

           251 ALIGLIIFCCRKKRREEKYEKEVHHDIREDVPPPKSRTSTARSYIGSNHS     300
               ||||||||||||||||||||||||||||||||||||||||||||||||||
           251 ALIGLIIFCCRKKRREEKYEKEVHHDIREDVPPPKSRTSTARSYIGSNHS     300

           301 SLGSMSPSNMEGYSKTQYNQVPSEDFERTPQSPTLPPAKFKYPY------     350
               ||||||||||||||||||||||||||||||||||||||   |
           301 SLGSMSPSNMEGYSKTQYNQVPSEDFERTPQSPTLPPAKVAAPNLSRMGA     350

           351 ---------KTDG-ITVV*...............................     400
                        | || | | |
           351 IPVMIPAQSK-DGSI-V-*...............................     400
```

Supplementary Figure 16. Amino acid sequences of CXADR isoforms. Amino acid sequences of CXADR (isoform 1) and CXADR iso5 (isoform 5) are shown. Underlined sequences are transmembrane domain.

Supplementary Figure 17

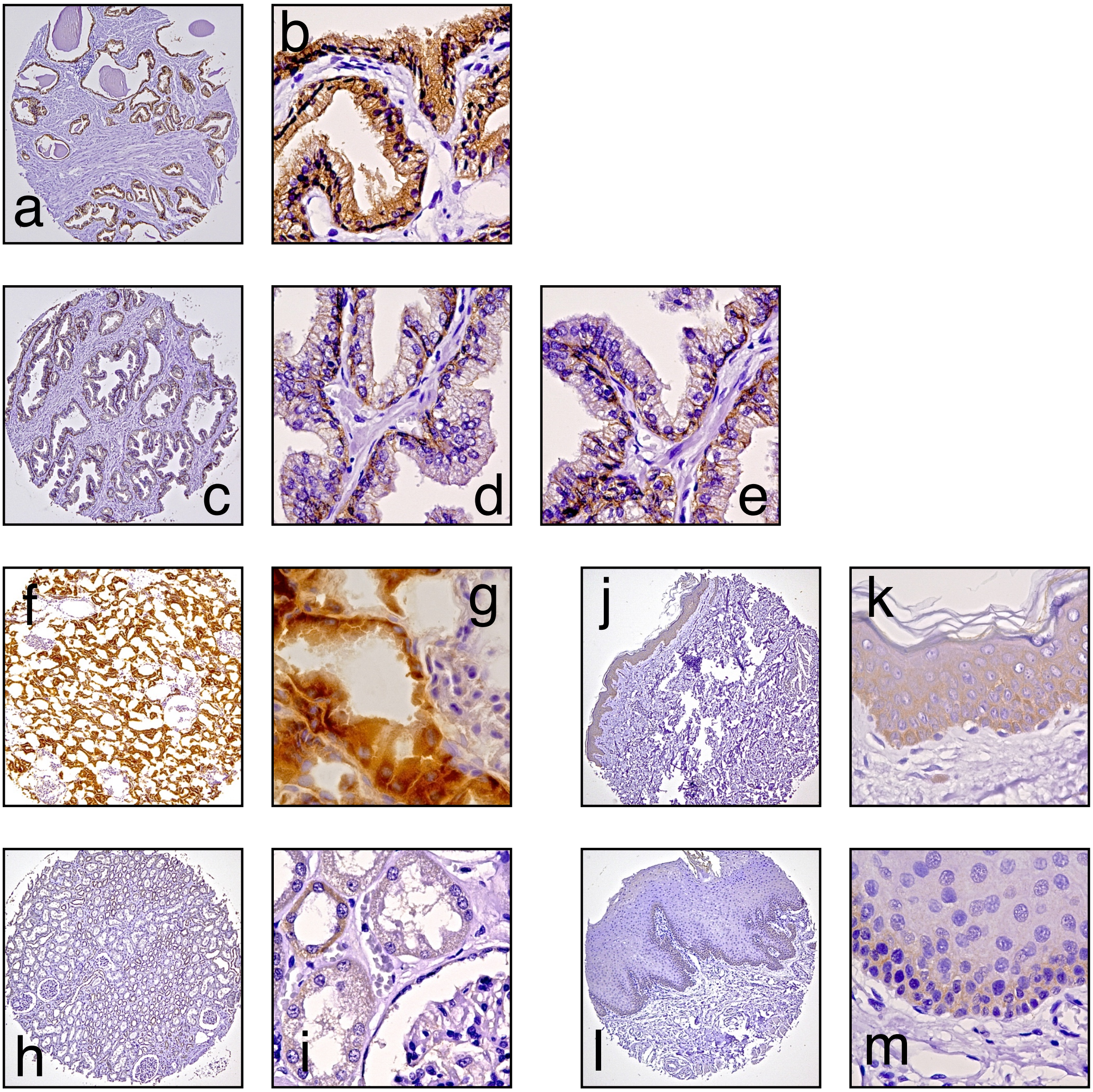

Supplementary Figure 17. Immunoreactivity of anti-CXADR antibody 6G10A on human tissues. Tissue sections (n=3) from prostate (a-e), kidney (f-i), and skin (j-m) were stained with anti-CXADR antibody 6G10A. Photos (b, d, e, g, i, k, m) are high magnification of photos (a, c, c, f, h, j, l), respectively.

Supplementary Figure 18

	CXADR antibody clone							
Antigen	2A8A	2A8B	3E8B	6G10A	7F8A	8B11B	8D6	8F11
1-83	−	−	−	−	−	−	−	−
1-133	−	−	−	−	−	−	−	−
1-181	−	−	−	−	+	−	−	−
1-230	+	+	+++	+++	+++	+++	+++	+
1-237	+++	+++	+++	+++	+++	+++	+++	+++

Supplementary Figure 18. Reactivity of anti-CXADR antibody clone against various CXADR mutants. Ba/F3 cells expressing various antigens of CXADR were constructed. Then, reactivity of anti-CXADR antibody clone against them was determined by FACS analysis.
